# Supplementary material for: Clusters explaining the relation between menopause and self-reported periodontal disease: a cross-sectional study
Source: PeerJ. 2025 Jan 27;13:e18861. doi: 10.7717/peerj.18861 (PMC11781263; doi:10.7717/peerj.18861)
Supplement: Supplemental Information 2 [file peerj-13-18861-s002.docx]

STROBE Statement—checklist of items that should be included in reports of observational studies

|  | Item No. | Recommendation | Page  No. | Relevant text from manuscript |
| --- | --- | --- | --- | --- |
| **Title and abstract** | 1 | (*a*) Indicate the study’s design with a commonly used term in the title or the abstract | 1 | **Lines 2-4:** “Clusters explaining the relation between menopause and self-reported periodontal “disease: A cross-sectional study |
|  |  | (*b*) Provide in the abstract an informative and balanced summary of what was done and what was found | 2 | **Lines 41-58:** **“Background**. Menopause is an important milestone in the women’s life continuum and is associated with potentially adverse effects, including those related to oral health. This study assessed self-reported periodontal disease in relation to menopausal status.  **Methods**. A cross-sectional study involving a convenience sample of female university dental hospital attendees was conducted using a validated, self-administered, self-reported periodontal disease questionnaire. A two-step cluster analysis was used to categorize the participants based on menstrual period (MP) continuity, systemic diseases and age. Differences between clusters were analyzed using chi-square test.  **Results**. From 112 included participants, 3 clusters resulted from the analysis: Cluster #1 (37±8 years, no systemic diseases and continued MP), Cluster #2 (40±10, with systemic diseases and continued MP) and Cluster #3 (54±9, with systemic diseases and discontinued MP). Cluster #3 had less optimal oral hygiene habits and more missing teeth (p>0.05). Clusters #1 and #2 reported more gingival bleeding, tooth sensitivity and calculus (p>0.05). Cluster #3, on the other hand, presented with more self-reported oral dryness (p≤0.05).  **Conclusions**. Within study limits, clusters of menopausal women with systemic diseases reported high symptoms of periodontal disease that were not significantly different from younger individuals, with the exception of oral dryness.” |
| Introduction | | | |  |
| Background/rationale | 2 | Explain the scientific background and rationale for the investigation being reported | 3 | **Lines 111-118:** “… Despite the available evidence, there are no data that have focused on the personal input of women in Northwest Saudia Arabia with regards to their oral health, namely periodontal disease and the possible relation with their menopausal condition. This can help oral health promotion, early treatment and prevention of further complications associated with different stages of menopause. Specifically, this will aid in the establishment of a referral system between gynecologists, general practitioners and dentists that involves regular dental checkups for early detection and intervention, and based on the fact that oral health is an integral component of the overall health.” |
| Objectives | 3 | State specific objectives, including any prespecified hypotheses | 4 | **Lines 120-122:** “… The objective of this study was thus to assess the self-reported periodontal disease among a selected group of women attending a university outpatient dental clinic in relation to their menopausal status.” |
| Methods | | | |  |
| Study design | 4 | Present key elements of study design early in the paper | 4 | **Line 126:** “… This was an observational cross-sectional analytical study …” |
| Setting | 5 | Describe the setting, locations, and relevant dates, including periods of recruitment, exposure, follow-up, and data collection | 4 | **Lines 126-127:** “… conducted at the Taibah University College of Dentistry outpatient clinics in AlMadinah AlMunawwarah, Saudi Arabia.”  **Lines 143-144:** “… who were attending the college clinics between November 2018 and July 2019.” |
| Participants | 6 | (*a*) *Cohort study*—Give the eligibility criteria, and the sources and methods of selection of participants. Describe methods of follow-up  *Case-control study*—Give the eligibility criteria, and the sources and methods of case ascertainment and control selection. Give the rationale for the choice of cases and controls  *Cross-sectional study*—Give the eligibility criteria, and the sources and methods of selection of participants | 4 | **Lines 142-145:** “… a convenience / consecutive sample from all female individuals aged between 22 and 75 years, who were attending the college clinics between November 2018 and July 2019. Children and individuals below 22 years were excluded alongside those with dementia or mental health or radiotherapy.” |
|  |  | (*b*) *Cohort study*—For matched studies, give matching criteria and number of exposed and unexposed  *Case-control study*—For matched studies, give matching criteria and the number of controls per case | - | - |
| Variables | 7 | Clearly define all outcomes, exposures, predictors, potential confounders, and effect modifiers. Give diagnostic criteria, if applicable | 4, 5 | **Line 155:** “… Data were collected by means of an interview and a self-administered questionnaire.”  **Lines 158-160:** “… included providing information about age, education level, marital stats, siblings, current health status and social and oral hygiene habits. Participants were also asked about continuation of their menstrual period (MP).”  **Lines 160-163:** “… a valid and reliable Arabic version of the self-reported periodontal health questionnaire was included. … This section consisted of seventeen close ended dichotomous questions, representing the outcome variables.” |
| Data sources/ measurement | 8* | For each variable of interest, give sources of data and details of methods of assessment (measurement). Describe comparability of assessment methods if there is more than one group | 5 | **Lines 160-163:** “… a valid and reliable Arabic version of the self-reported periodontal health questionnaire was included. … This section consisted of seventeen close ended dichotomous questions, representing the outcome variables.” |
| Bias | 9 | Describe any efforts to address potential sources of bias | 4 | As publication bias is more pronounced when sample size is small, a sample size calculation was conducted to assure sufficient sample size.  **Lines 148-151:** “…Based on the reported prevalence of the outcome “gingivitis” in the general population of 75%, with a desired level of confidence of 95%, and a margin of error of 10, and an estimated population of women attending the clinics annually of 6,500, a sample size of 97 participants was required for the study.” |
| Study size | 10 | Explain how the study size was arrived at | 4 | **Lines 148-151:** “…Based on the reported prevalence of the outcome “gingivitis” in the general population of 75%, with a desired level of confidence of 95%, and a margin of error of 10, and an estimated population of women attending the clinics annually of 6,500, a sample size of 97 participants was required for the study.” |

Continued on next page

| Quantitative variables | 11 | Explain how quantitative variables were handled in the analyses. If applicable, describe which groupings were chosen and why | 5 | **Line 166:** “…Means and standard deviations were used to describe continuous variables …” |
| --- | --- | --- | --- | --- |
| Statistical methods | 12 | (*a*) Describe all statistical methods, including those used to control for confounding | 5 | **Lines 177-180:** “…The differences between groups/clusters in terms of quantitative demographics, oral hygiene habits, oral findings and the self-reported periodontal disease questions were analyzed using chi-square test. A p-Value ≤ 0.05 was considered statistically significant.” |
|  |  | (*b*) Describe any methods used to examine subgroups and interactions | 5 | **Lines 173-175:** “…a 2-step cluster analysis was performed in order to identify hidden patterns and relationships by categorizing the participants based on the available background, demographic and health data.” |
|  |  | (*c*) Explain how missing data were addressed | 4 | **Line 152:** “…Incomplete responses were to be excluded from the analysis.” |
|  |  | (*d*) *Cohort study*—If applicable, explain how loss to follow-up was addressed  *Case-control study*—If applicable, explain how matching of cases and controls was addressed  *Cross-sectional study*—If applicable, describe analytical methods taking account of sampling strategy | 5 | **Lines 177-180:** “…The differences between groups/clusters in terms of quantitative demographics, oral hygiene habits, oral findings and the self-reported periodontal disease questions were analyzed using chi-square test. A p-Value ≤ 0.05 was considered statistically significant.” |
|  |  | (*e*) Describe any sensitivity analyses | 5 | **Line 180:** “…A p-Value ≤ 0.05 was considered statistically significant.” |
| Results | | | | |
| Participants | 13* | (a) Report numbers of individuals at each stage of study—eg numbers potentially eligible, examined for eligibility, confirmed eligible, included in the study, completing follow-up, and analysed | 5 | **Line 184:** “…Of a total of 114 participating female patients…” **Line 185:** “…112 returned their completed questionnaires…”  **Lines 194-196:** “…cluster # 1 (n=65) including women with no systemic conditions and cluster # 3 (n=22) involving those with a completely discontinued menstrual period (Figure 1)”. |
|  |  | (b) Give reasons for non-participation at each stage | 5 | **Line 184:** “…two were excluded due to incomplete data.” |
|  |  | (c) Consider use of a flow diagram | - | - |
| Descriptive data | 14* | (a) Give characteristics of study participants (eg demographic, clinical, social) and information on exposures and potential confounders | 5 | **Lines 186-192:** “…The mean age of the total sample was 40 (±10) years. Fifty-two percent held a university degree and 66% were employed. Thirty-six percent of the participants suffered from different systemic conditions such as hypertension and diabetes mellitus. Similarly, 59% were on chronic medication, five of which specifically reporting the use of hormone replacement therapy (HRT). Fourteen percent of the total participants were current smokers, 3 of them smoked cigarettes and the remaining used waterpipes. Twenty percent had already discontinued their menstrual period.” |
|  |  | (b) Indicate number of participants with missing data for each variable of interest | 5 | **Line 184:** “…two were excluded due to incomplete data.” |
|  |  | (c) *Cohort study*—Summarise follow-up time (eg, average and total amount) | - | - |
| Outcome data | 15* | *Cohort study*—Report numbers of outcome events or summary measures over time | - | - |
|  |  | *Case-control study—*Report numbers in each exposure category, or summary measures of exposure | - | - |
|  |  | *Cross-sectional study—*Report numbers of outcome events or summary measures | 5 | **Lines 162-163:** “… This section consisted of seventeen close ended dichotomous questions, representing the outcome variables.” |
| Main results | 16 | (*a*) Give unadjusted estimates and, if applicable, confounder-adjusted estimates and their precision (eg, 95% confidence interval). Make clear which confounders were adjusted for and why they were included | - | - |
|  |  | (*b*) Report category boundaries when continuous variables were categorized | 5 | **Lines 167-171:** “… For simplicity during data presentation, the different chronic illnesses that the participants were suffering from were grouped under one category, i.e. systemic conditions, since most were considered of inflammatory nature. Similarly, different long-term medications were grouped together, acknowledging their negative effect on salivary secretion.” |
|  |  | (*c*) If relevant, consider translating estimates of relative risk into absolute risk for a meaningful time period | - | - |

Continued on next page

| Other analyses | 17 | Report other analyses done—eg analyses of subgroups and interactions, and sensitivity analyses | 5 | **Lines 173-175:** “…a 2-step cluster analysis was performed in order to identify hidden patterns and relationships by categorizing the participants based on the available background, demographic and health data.” |
| --- | --- | --- | --- | --- |
| Discussion | | | | |
| Key results | 18 | Summarise key results with reference to study objectives | 6 | **Lines 216-218:** “… This study aimed at evaluating self-reported oral health in women in relation to their menopausal status. Women who have not reached menopausal age insignificantly reported more bleeding gums, tooth sensitivity and calculus deposits than their menopausal counterparts.” |
| Limitations | 19 | Discuss limitations of the study, taking into account sources of potential bias or imprecision. Discuss both direction and magnitude of any potential bias | 7 | **Lines 254-261:** “…The relatively small sample size is looked upon as a study limitations, since it may have impacted the extrapolation of the observed findings alongside the convenience sampling that precluded generalizability of the finding. This may dictate the consideration of the current investigation as a pilot study at best. However, the self-reporting of oral health symptoms in menopausal women in this study covers an important aspect, providing unique information that can be utilized for hypothesis generation in future studies. Moreover, the cross-sectional design limits the identification of temporal relationships between menopause and oral health parameters, necessitating the interpretation of findings with caution.” |
| Interpretation | 20 | Give a cautious overall interpretation of results considering objectives, limitations, multiplicity of analyses, results from similar studies, and other relevant evidence | 7 | **Lines 259-261:** “…the cross-sectional design limits the identification of temporal relationships between menopause and oral health parameters, necessitating the interpretation of findings with caution.” |
| Generalisability | 21 | Discuss the generalisability (external validity) of the study results | 7 | **Lines 254-256:** “…The relatively small sample size is looked upon as a study limitations, since it may have impacted the extrapolation of the observed findings alongside the convenience sampling that precluded generalizability of the finding.” |
| Other information | |  | | |
| Funding | 22 | Give the source of funding and the role of the funders for the present study and, if applicable, for the original study on which the present article is based | - | - |

*Give information separately for cases and controls in case-control studies and, if applicable, for exposed and unexposed groups in cohort and cross-sectional studies.

**Note:** An Explanation and Elaboration article discusses each checklist item and gives methodological background and published examples of transparent reporting. The STROBE checklist is best used in conjunction with this article (freely available on the Web sites of PLoS Medicine at http://www.plosmedicine.org/, Annals of Internal Medicine at http://www.annals.org/, and Epidemiology at http://www.epidem.com/). Information on the STROBE Initiative is available at www.strobe-statement.org.
